# Therapeutic Potential of Berberine in Obesity-Associated Neuroinflammation Through Shared Molecular Pathways: TLR4/NF-κB/MAPK, ROS/NRF2 and NLRP3

**DOI:** 10.3390/nu18183000

**Published:** 2026-09-14

**Authors:** María Del Refugio Moyetón-Hernández, Cindy Bandala, Mariana Guadarrama-Castillo, Roberto Medina-Santillán, Eleazar Lara-Padilla

**Affiliations:** 1 Sección de Estudios de Posgrado de Investigación, Escuela Superior de Medicina, Instituto Politécnico Nacional, Mexico City 11340, Mexico; mmoyetonh1700@alumno.ipn.mx (M.D.R.M.-H.); rmedinas@ipn.mx (R.M.-S.); 2 Laboratorio de Neurociencia Traslacional, Escuela Superior de Medicina, Instituto Politécnico Nacional, Mexico City 11340, Mexico; crodriguezba@ipn.mx; 3 Laboratorio de Enfermedades Metabólicas, Escuela Superior de Medicina, Instituto Politécnico Nacional, Mexico City 11340, Mexico; mguadarramac1600@alumno.ipn.mx

**Keywords:** berberine, neuroinflammation, obesity, blood–brain barrier, TLR4, NF-κB, MAPK, reactive oxygen species, NRF2, NLRP3 inflammasome

## Abstract

Obesity is recognized as a pathological condition that induces chronic low-grade systemic inflammation capable of affecting multiple organs, including the central nervous system, thereby promoting neuroinflammation. Although adipose and neural tissues differ in their structural and functional characteristics, they share common inflammatory mechanisms involving the TLR4/NF-κB/MAPK signaling pathways, the ROS/NRF2 axis, and the NLRP3 inflammasome. This review summarizes current evidence demonstrating the ability of berberine (BBR) to modulate these shared molecular pathways across different experimental models and pathophysiological conditions, with particular emphasis on obesity-induced neuroinflammation. A narrative literature search was conducted using academic search and indexing resources, including PubMed, Scopus, Web of Science, and Google Scholar, with the literature updated through 24 August 2026. The available evidence indicates that BBR modulates these signaling pathways, reduces the production of pro-inflammatory cytokines, attenuates oxidative stress, and limits glial activation. Furthermore, several studies suggest that BBR may help preserve the functional integrity of the blood–brain barrier and reduce neuronal damage associated with neuroinflammatory processes. Available evidence suggests that BBR may modulate inflammatory and oxidative pathways involved in obesity-associated neuroinflammation; however, current findings derive mainly from preclinical studies and indirect models. Future studies are needed to determine the bioavailability, central nervous system penetration, and clinical relevance of BBR.

## 1. Introduction

Obesity is a highly prevalent chronic disease worldwide [1] and increases the risk of developing several comorbidities, including cardiovascular disease, type 2 diabetes mellitus (T2DM), metabolic dysfunction-associated steatotic liver disease (MASLD), obstructive sleep apnea, and neurological disorders, among others [2,3]. The pathophysiological link between obesity and multi-organ damage is largely driven by chronic low-grade systemic inflammation [4]. This persistent inflammatory state also affects the central nervous system (CNS), promoting chronic neuroinflammation that increases the risk of cognitive impairment and neurodegenerative disorders such as Alzheimer’s disease and dementia [5].

Evidence indicates that inflammatory mechanisms operating in adipose tissue and the CNS converge through several shared molecular pathways, particularly the Toll-like receptor 4 (TLR4)/nuclear factor kappa B (NF-κB), mitogen-activated protein kinase (MAPK), reactive oxygen species (ROS)/nuclear factor erythroid 2-related factor 2 (NRF2), and NOD-, LRR-, and pyrin domain-containing protein 3 (NLRP3) inflammasome pathways [6,7,8,9,10,11]. These signaling cascades represent relevant molecular targets for developing potential strategies to modulate obesity-associated neuroinflammation. A wide number of therapeutic strategies, including bariatric surgery, pharmacological interventions, and bioactive compounds, have been shown to attenuate neuroinflammation by reducing systemic inflammation associated with obesity [12]. However, although both surgical and pharmacological treatments have demonstrated clinical benefits, they also present important limitations [13]. Consequently, naturally occurring bioactive compounds have attracted increasing interest as potential modulators of molecular pathways involved in obesity and its associated neuroinflammatory processes [12,13,14,15]. Among these compounds, berberine (BBR) has attracted considerable attention because of its potential to modulate the molecular pathways implicated in obesity and neuroinflammation [16].

BBR is an isoquinoline alkaloid naturally found in several plant species, particularly those belonging to the *Berberidaceae* family. It has been used for more than 3000 years in traditional medicine systems, especially Traditional Chinese Medicine and Ayurveda [17,18]. Extensive experimental and clinical evidence has demonstrated that BBR exhibits a broad range of pharmacological activities, including anti-inflammatory, antioxidant, immunomodulatory, antimicrobial, hypoglycemic, hypolipidemic, cardioprotective, neuroprotective, and anticancer effects [17,18,19,20,21]. Accordingly, BBR has been investigated as a potential therapeutic agent for numerous pathological conditions, including metabolic, cardiovascular, autoimmune, neoplastic, and infectious diseases [17,18]. In addition, increasing evidence supports its therapeutic potential in neurodegenerative disorders such as Alzheimer’s, Parkinson’s, and Huntington’s diseases, as well as in neuropsychiatric conditions including depression and anxiety [19,20,21,22].

Therefore, this narrative review summarizes and integrates the current evidence regarding the direct and indirect mechanisms by which BBR may attenuate obesity-associated neuroinflammation, with particular emphasis on the TLR4/NF-κB, MAPK, ROS/NRF2, and NLRP3 signaling pathways.

## 2. Materials and Methods

A narrative literature review was conducted to integrate relevant evidence around two main axes: (1) the pathophysiological mechanisms involved in obesity, low-grade systemic inflammation, and neuroinflammation; and (2) the potential therapeutic targets of BBR on molecular pathways shared between metabolic inflammation and neuroinflammation, including TLR4/NF-κB, MAPK, ROS/NRF2, and NLRP3.

Academic search and indexing resources, including PubMed, Scopus, Web of Science, and Google Scholar, were used to identify potentially relevant literature. Individual terms and combinations adapted to the thematic block evaluated were used, including “obesity,” “adipose tissue inflammation,” “metaflammation,” “metabolic inflammation,” “neuroinflammation,” “blood–brain barrier,” “oxidative stress,” “reactive oxygen species,” “TLR4,” “NF-κB,” “MAPK,” “NRF2,” “NLRP3 inflammasome,” “microglia,” “astrocytes,” “pericytes,” “AMPK,” “berberine,” and “neuroprotection.” Depending on the mechanism evaluated, thematic combinations of these terms were used, for example, “obesity” + “neuroinflammation,” “obesity” + “blood–brain barrier,” “berberine” + “neuroinflammation,” “berberine” + “TLR4,” “berberine” + “NF-κB,” “berberine” + “NRF2,” and “berberine” + “NLRP3.”

The inclusion criteria were as follows: (a) original preclinical and clinical studies, as well as narrative and systematic reviews, published between 1993 and 2026; and (b) studies providing evidence relevant to the following thematic domains of the review: (i) obesity-associated low-grade systemic inflammation and adipose tissue inflammation; (ii) obesity-related neuroinflammation and blood–brain barrier dysfunction; (iii) TLR4/NF-κB/MAPK, ROS/NRF2, or NLRP3/autophagy signaling; (iv) biological effects of BBR on these pathways in metabolic, inflammatory, or CNS models; or (v) BBR pharmacokinetics, bioavailability, brain distribution, or human obesity-related outcomes. Studies were excluded when they did not provide mechanistic, pathophysiological, pharmacokinetic, or clinical information relevant to these thematic domains. Because direct studies of BBR in obesity-associated neuroinflammation are scarce, etiologically distinct metabolic and CNS models were retained when they interrogated mechanistically overlapping pathways; these studies were treated as indirect evidence. As this was a narrative review, study selection was based on thematic relevance rather than on a formal systematic-review screening protocol.

## 3. Obesity as a Trigger of Chronic Low-Grade Systemic Inflammation

Adipose tissue exhibits distinct physiological and pathophysiological characteristics depending on its anatomical location [23,24]. Visceral adipose tissue (VAT) contains a lower proportion of preadipocytes and a greater number of hypertrophic adipocytes, a phenotype that has been consistently associated with a systemic pro-inflammatory state [25,26,27]. Adipocyte hypertrophy increases the oxygen diffusion distance while reducing functional capillary density, thereby promoting adipose tissue hypoxia [28,29,30,31]. Within this hypoxic microenvironment, stabilization of hypoxia-inducible factor-1 alpha (HIF-1α) has been associated with the activation of pro-inflammatory signaling pathways, including nuclear factor kappa B (NF-κB), leading to the increased production of cytokines such as tumor necrosis factor-alpha (TNF-α), interleukin-6 (IL-6), and monocyte chemoattractant protein-1 (MCP-1) [30]. Moreover, experimental studies have demonstrated that reduced oxygen availability promotes the polarization of macrophages from the anti-inflammatory M2 phenotype toward the pro-inflammatory M1 phenotype [32]. M1 macrophages produce elevated levels of TNF-α, interleukin-1 beta (IL-1β), and IL-6, thereby amplifying adipose tissue inflammation [33,34].

Inflammatory signaling within adipose tissue may be triggered through both hypoxia-dependent and hypoxia-independent mechanisms [31]. However, the role of hypoxia in obesity remains controversial. Recent evidence has reported the presence of adipose tissue hyperoxia in humans, suggesting that increased oxygen tension may result from mitochondrial dysfunction and a consequent reduction in oxygen consumption rather than enhanced oxygen delivery [35]. Collectively, these findings indicate that obesity is characterized by altered adipose tissue oxygenation, which promotes the activation of pro-inflammatory pathways involved in both local and systemic inflammatory responses [35,36,37,38].

Another hallmark of obesity is increased oxidative stress [38]. Lipotoxicity induced by saturated fatty acids has been shown to enhance the generation of reactive oxygen species (ROS) through activation of nicotinamide adenine dinucleotide phosphate (NADPH) oxidase and mitochondrial dysfunction [39,40]. In an in vitro study using differentiated 3T3-L1 murine adipocytes, palmitic acid induced endoplasmic reticulum stress accompanied by apoptosis, cellular dysfunction, and increased ROS production [41]. These reactive species act as important mediators of NLRP3 inflammasome activation, promoting caspase-1 activation and the subsequent release of IL-1β [42]. Nevertheless, ROS are not the only mechanism involved in NLRP3 activation. Several metabolic stimuli, including saturated fatty acids (SFAs), can promote inflammatory responses involving pattern recognition receptors such as Toll-like receptor 2 (TLR2) and Toll-like receptor 4 (TLR4), thereby favoring NF-κB activation [42,43,44,45]. In this context, TLR4-dependent signaling contributes to the induction of pro-inflammatory mediators such as TNF-α, IL-6, and pro-IL-1β, as well as the expression of NLRP3 inflammasome components, thus amplifying the inflammatory response [46].

In response to increased ROS production, cells activate antioxidant defense mechanisms primarily through nuclear factor erythroid 2-related factor 2 (NRF2) [47,48]. Activation of NRF2 induces the expression of antioxidant enzymes, including superoxide dismutase (SOD), catalase, glutathione peroxidase (GPx), and heme oxygenase-1 (HO-1) [49]. In addition to its antioxidant functions, NRF2 has been shown to suppress both NLRP3 inflammasome activation [50,51] and NF-κB signaling [52], thereby reducing the production of pro-inflammatory cytokines [53]. In obesity, impairment of the NRF2 signaling pathway weakens the antioxidant response, contributing to redox imbalance and perpetuating oxidative stress and inflammation [48,49].

## 4. Pathophysiological Mechanisms of Neuroinflammation in Obesity

**Figure 1:**
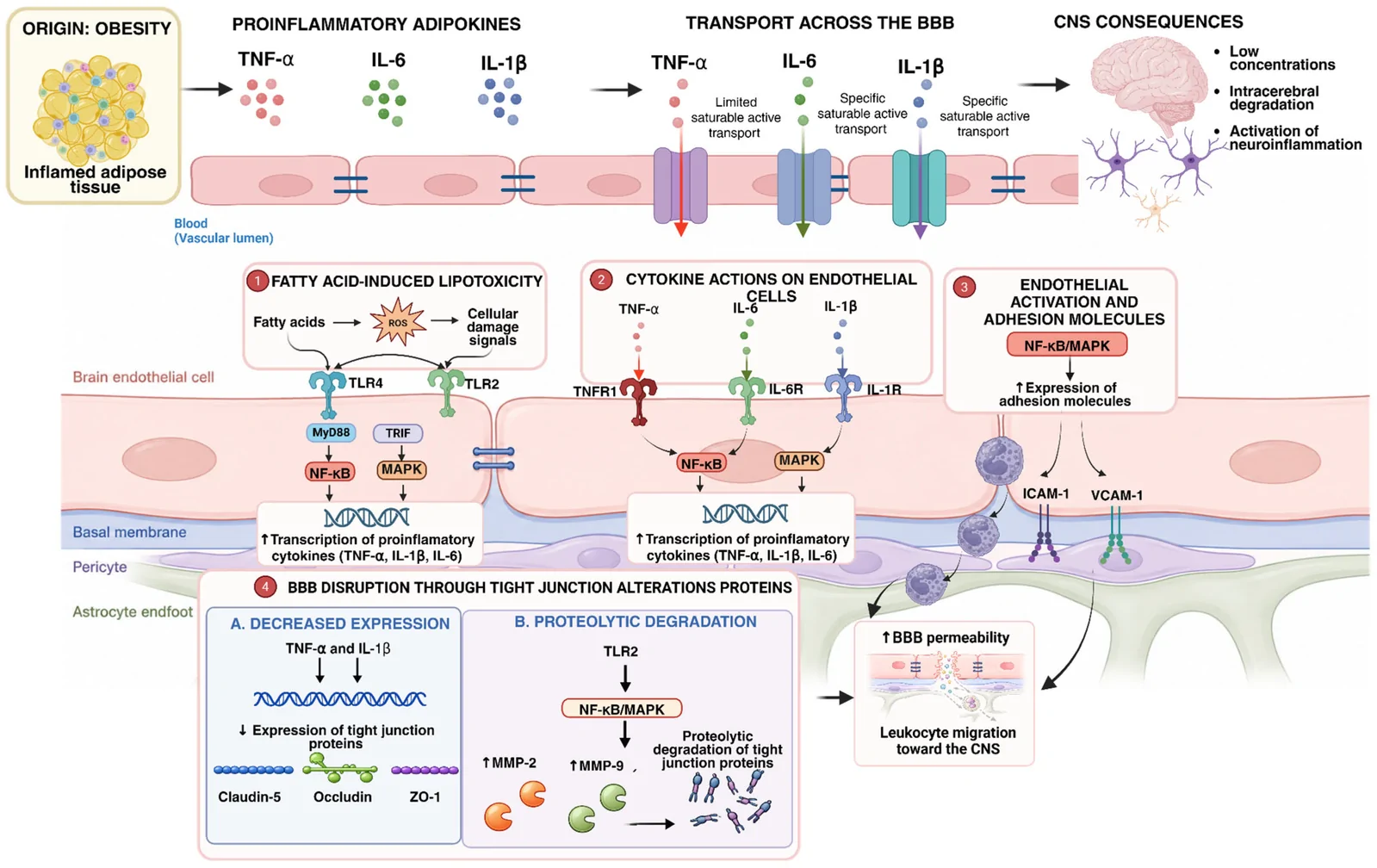
Molecular mechanisms involved in blood–brain barrier disruption during obesity-associated neuroinflammation. Obesity induces chronic low-grade systemic inflammation characterized by increased production of pro-inflammatory mediators in adipose tissue. Selected circulating cytokines can undergo regulated transport from the blood to the brain; however, transport efficiency varies among mediators, and much of the supporting evidence comes from experimental studies in rodents. TNF-α exhibits limited, saturable blood-to-brain transport and, in obesity, adipose tissue-derived TNF-α acts predominantly through local autocrine and paracrine mechanisms. Importantly, the endothelial effects of circulating TNF-α illustrated in this figure do not require substantial passage across the BBB, as TNF-α can activate TNF receptors on the luminal surface of brain endothelial cells, thereby initiating intracellular inflammatory signaling. (**1**) Saturated fatty acids promote endothelial lipotoxicity, ROS generation, and cellular damage signaling associated with the activation of TLR4- and TLR2-related inflammatory pathways. (**2**) Circulating inflammatory cytokines interact with their corresponding receptors on brain endothelial cells, activating NF-κB and MAPK signaling and increasing the production of pro-inflammatory mediators. In the case of TNF-α, this mechanism involves endothelial receptor signaling from the vascular compartment and does not require substantial trans-BBB entry. (**3**) Activation of NF-κB and MAPK signaling promotes the expression of adhesion molecules, including ICAM-1 and VCAM-1, thereby facilitating leukocyte adhesion and migration toward the CNS. (**4**) BBB dysfunction is associated with alterations in tight-junction proteins through two major mechanisms: (**A**) decreased expression induced by inflammatory cytokine signaling, including signaling by TNF-α and IL-1β in BBB-associated cells, and (**B**) increased proteolytic degradation, particularly through MMP-2 and MMP-9. Once neuroinflammation is established, TNF-α produced locally by activated microglia and reactive astrocytes may further amplify inflammatory signaling within the CNS; this local glial source is not depicted in the figure.

**Figure 2:**
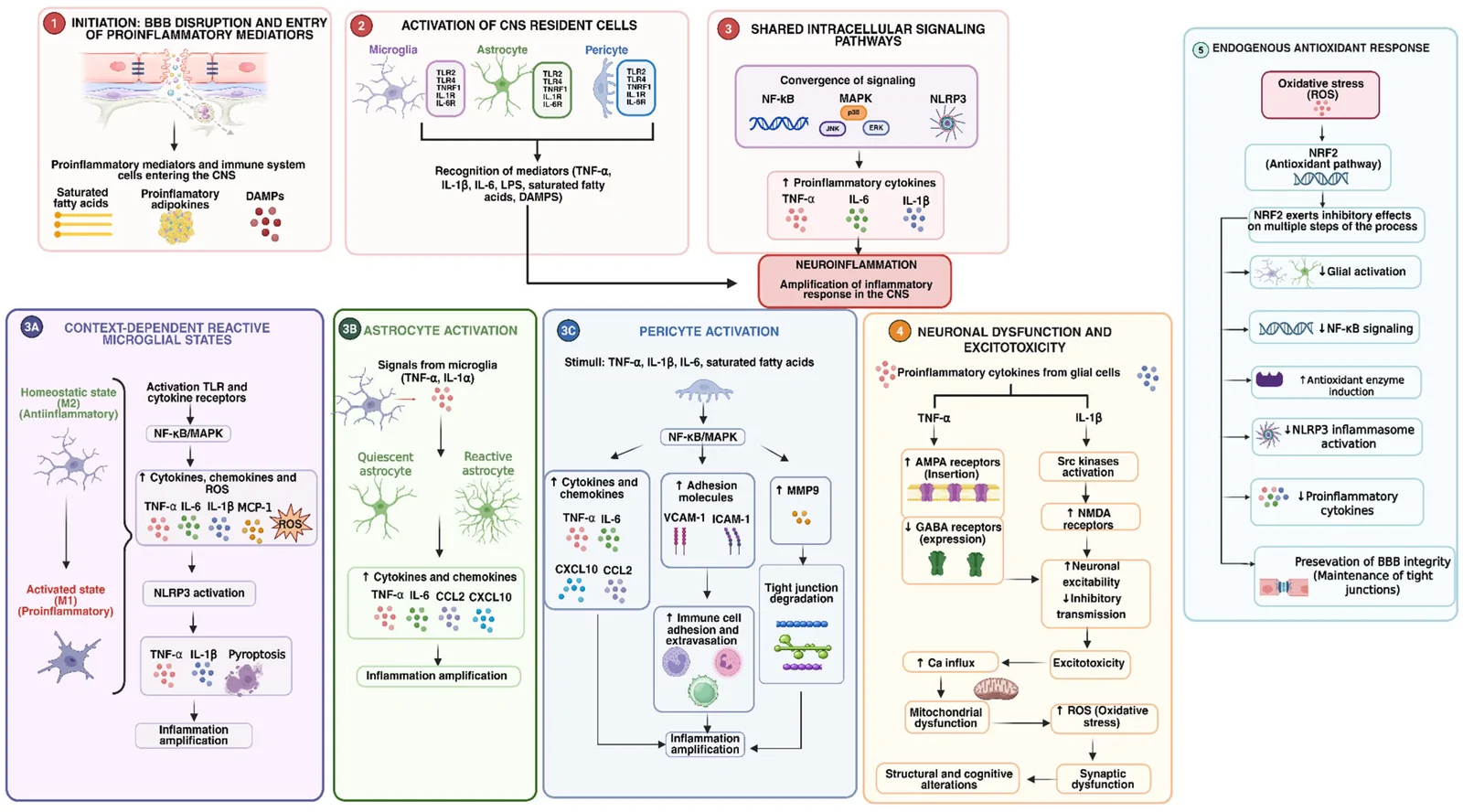
Molecular mechanisms involved in obesity-associated neuroinflammation. (**1**) Following blood–brain barrier (BBB) disruption, pro-inflammatory mediators, saturated fatty acids, and damage-associated molecular patterns (DAMPs) cross the BBB. (**2**) These mediators interact with receptors shared by microglia, astrocytes, and pericytes, including TLR2, TLR4, TNFR1, IL-1R, and IL-6R. (**3**) Ligand binding to these receptors induces activation of the NF-κB, MAPK, and NLRP3 signaling pathways, promoting the production of pro-inflammatory cytokines and amplifying neuroinflammation. (**3A**) In microglia, inflammatory stimuli induce context-dependent reactive states characterized by activation of the NF-κB, MAPK, and NLRP3 pathways, increased production of pro-inflammatory mediators, and pyroptosis. (**3B**) Astrocytes are stimulated by these inflammatory mediators, particularly TNF-α and IL-1α, inducing a transition from a quiescent to a reactive state and thereby contributing to the amplification of inflammation. (**3C**) These mediators also activate pericytes, which not only intensify the inflammatory response but also increase the expression of adhesion molecules and MMP-9, further promoting BBB disruption and the infiltration of inflammatory cells. (**4**) Neurons are also affected by this inflammatory microenvironment. TNF-α increases the insertion of AMPA receptors into the plasma membrane and reduces GABA receptor expression, thereby enhancing neuronal excitability and promoting excitotoxicity. IL-1β also contributes to excitotoxicity by increasing NMDA receptor activity through Src kinase activation. The resulting increase in intracellular calcium influx is accompanied by mitochondrial dysfunction, increased reactive oxygen species (ROS) production, and synaptic impairment, ultimately leading to structural alterations and cognitive dysfunction. (**5**) Concurrently, NRF2 signaling is activated as a compensatory response to oxidative and inflammatory stimuli. NRF2 activation reduces glial activation, NF-κB signaling, pro-inflammatory cytokine production, and NLRP3 inflammasome activation, while increasing the expression of antioxidant enzymes and contributing to the preservation of BBB integrity.

Neuroinflammation is defined as the compensatory inflammatory response of the central nervous system (CNS) to tissue injury or danger signals. It is characterized by the activation of glial cells, primarily microglia and astrocytes, as well as other components of the neurovascular unit, including pericytes [54,55]. This response may be triggered by infections, traumatic injury, toxins, systemic inflammatory states, and other stimuli, leading to the production of pro-inflammatory mediators within the CNS [56]. Two major forms of neuroinflammation have been described: acute and chronic. Acute neuroinflammation is typically associated with brain injury, trauma, and CNS infections, whereas chronic neuroinflammation may arise from unresolved acute inflammation or prolonged exposure to damage-associated molecular patterns (DAMPs) [57], which are commonly linked to the pathophysiological processes underlying obesity and its associated comorbidities [5]. Experimental and clinical evidence has demonstrated that adipokines can reach multiple organs through circulation, including the CNS [37]. Several mechanisms have been proposed through which these adipokines cross the blood–brain barrier (BBB) and promote neuroinflammation (Figure 1 and Figure 2) [58,59].

The BBB is a highly selective structure that regulates the exchange of molecules between the systemic circulation and the CNS. It is primarily composed of endothelial cells, pericytes, and astrocytes [58]. Endothelial cells play a central role in controlling molecular transport across the BBB through multiple transport mechanisms. Unlike peripheral capillaries, BBB endothelial cells lack fenestrations [58,59], and their permeability is largely determined by tight junction proteins, including occludins, claudins (claudin-1, -3, -4, -5, and -12), and membrane-associated guanylate kinase proteins such as zonula occludens-1 (ZO-1), ZO-2, and ZO-3) [58,60]. In addition, BBB endothelial cells express receptors for TNF-α, IL-1β, IL-6, and Toll-like receptors (TLR2, TLR3, TLR4, TLR6, and TLR9) [61,62,63].

Although small lipophilic molecules may cross the BBB by passive diffusion, most substances require specialized transport systems, including solute carriers and receptor-mediated transcytosis [59]. Pro-inflammatory cytokines can influence neuroinflammatory signaling through several mechanisms involving the BBB, including the activation of endothelial receptors and, in the case of some cytokines, regulated transport from the blood to the brain [61,62,63,64,65,66,67,68]. Experimental studies in mice have demonstrated that the transport of TNF-α, IL-6, and IL-1β across the BBB is saturable [65,66,67,68]. However, the magnitude of transport varies among cytokines and may be modified by physiological and pathological conditions. Importantly, the effects of TNF-α on the blood–brain barrier (BBB) must be distinguished according to its origin and site of action. In obesity, adipose tissue-derived TNF-α acts predominantly through local autocrine and paracrine mechanisms, and its circulating concentrations are relatively low [69,70,71]. Although saturable and limited transport of TNF-α from the blood to the brain has been demonstrated in rodent models [65,66], substantial entry into the brain parenchyma is not required for peripheral TNF-α to influence the CNS. Brain endothelial cells express TNF receptors and can respond to circulating TNF-α at the luminal surface, activating inflammatory signaling and altering barrier function [61,62,72,73]. Once neuroinflammation is established, TNF-α produced locally by activated microglia and reactive astrocytes can further amplify inflammatory signaling within the CNS [74,75,76,77].

IL-6 and IL-1β have also been shown to undergo saturable transport across the BBB in, although CNS exposure remains limited and is influenced by degradation and other regulatory processes mice [67,68]. Therefore, these experimental findings demonstrate that regulated cytokine transport across the BBB occurs, but they should not be quantitatively extrapolated to humans with obesity-associated inflammation.

Despite these low CNS concentrations, IL-1β exerts potent pro-inflammatory effects by binding to interleukin-1 receptor type 1 (IL-1R1) expressed on endothelial cells [78]. This interaction activates intracellular signaling pathways, primarily NF-κB and mitogen-activated protein kinase (MAPK), promoting the transcription of pro-IL-1β and NLRP3 inflammasome components, thereby contributing to neuroinflammation [78,79]. Therefore, the biological effects of circulating inflammatory mediators depend not only on their passage across the BBB but also on the downstream signaling cascades they activate, which ultimately induce transcriptional programs associated with neuroinflammation [62,67,68].

Evidence suggests that chronic exposure of the BBB to saturated fatty acids induces endothelial lipotoxicity [80]. This process promotes ROS generation and subsequent cellular damage signaling [81,82] leading to inflammatory signaling involving Toll-like receptors, particularly TLR4. TLR4 signaling proceeds through both the MyD88 (myeloid differentiation primary response protein 88)-dependent and TRIF (TIR-domain-containing adapter-inducing interferon-β)-dependent pathways, culminating in activation of NF-κB and MAPK signaling and increased production of TNF-α, IL-6, and IL-1β [63,81,83]. These locally generated and circulating inflammatory signals can induce endothelial activation and increase the expression of adhesion molecules such as ICAM-1 and VCAM-1, thereby increasing blood–brain barrier (BBB) permeability and facilitating leukocyte infiltration into the CNS [84].

BBB integrity may also be compromised through alterations in tight junction proteins, either by reduced expression or enhanced proteolytic degradation [65]. Experimental studies have demonstrated that TNF-α and IL-1β decrease the expression of claudin-5, occludin, and ZO-1 [72,73,85]. In addition, the degradation of tight junction proteins is mediated by matrix metalloproteinases (MMPs), a family of endopeptidases involved in extracellular matrix remodeling [86,87]. These enzymes are secreted by endothelial cells, leukocytes, microglia, reactive astrocytes, and neurons [87]. Under conditions of metabolic stress and inflammation, such as obesity, increased expression of MMP-2 and MMP-9 has been consistently observed [86]. Experimental studies have further demonstrated that TLR2 activation, as well as stimulation by TNF-α, lipopolysaccharide (LPS), and palmitic acid, enhances MMP-2 and MMP-9 expression through the MAPK/NF-κB signaling pathways, thereby promoting degradation of tight junction proteins and increasing BBB permeability [87,88,89,90,91].

Following BBB disruption, pro-inflammatory mediators—including adipokines, lipopolysaccharides, and saturated fatty acids—can act as DAMPs and activate multiple cell types within the CNS, particularly microglia, astrocytes, and pericytes, thereby amplifying the neuroinflammatory response [57]. Although these cell populations differ structurally and functionally, they share common inflammatory sensing mechanisms [92]. Notably, all express pattern recognition receptors, especially TLR2 and TLR4, as well as receptors for pro-inflammatory cytokines including IL-1R, TNFR, and IL-6R [74,92,93,94]. Consequently, these cells respond to multiple inflammatory stimuli derived from systemic inflammation, including TNF-α, IL-1β, IL-6, LPS, saturated fatty acids, advanced glycation end products, and DAMPs [74,94]. Activation of these receptors leads to convergence of intracellular signaling through the NF-κB, MAPK, and NLRP3 pathways [74,92,93,95,96] resulting in sustained production of TNF-α, IL-1β, and IL-6 [74,75,92,96].

Microglia are the main population of resident immune cells involved in CNS homeostasis [97]. Rather than switching between two distinct phenotypes, M1 and M2, microglia can adopt heterogeneous, dynamic, and context-dependent functional states in response to metabolic and inflammatory stimuli [98]. M1/M2 terminology is a simplified experimental framework rather than two mutually exclusive microglial phenotypes in vivo. Nevertheless, under pro-inflammatory conditions, microglial states may exhibit increased activation of signaling pathways related to NF-κB, MAPK, JAK/STAT, and the inflammasome, together with increased production of pro-inflammatory cytokines and ROS [92,99].

One relevant inflammatory condition is the excess of saturated fatty acids, as it can promote TLR4-dependent inflammatory signaling in hypothalamic microglia, contributing to NF-κB activation and increased production of TNF-α, IL-6, MCP-1, and IL-1β [94,100,101]. Although saturated fatty acids, such as palmitate, should not be considered direct TLR4 agonists, experimental evidence indicates that TLR4-dependent sensitization and the subsequent metabolic, transcriptional, and membrane lipid reprogramming create a cellular state that facilitates saturated fatty acid-induced inflammatory responses [102].

In addition to NF-κB, downstream MAPK mediators may further amplify neuroinflammation. For example, MAPK-activated protein kinase 2 (MK2), a downstream effector of p38 MAPK, has been shown to regulate inflammatory cytokine production. Experimental MK2 deficiency in activated microglia reduced TNF-α release, inflammatory chemokine production, and microglia-mediated neurotoxicity [103]. Sustained microglial activation has also been associated with the activation of the NLRP3 inflammasome, increased production of IL-1β and IL-18, and induction of pyroptosis through membrane pore formation, thereby amplifying neuroinflammation [95].

Microglia-derived TNF-α and IL-1α promote astrocyte activation through TNFR and IL-1R signaling [75]. Activated astrocytes subsequently acquire a reactive phenotype characterized by increased secretion of IL-6, TNF-α, and chemokines including CCL2 and CXCL10, further amplifying neuroinflammation [74,75,76,77].

Pericytes also play a fundamental role in maintaining BBB integrity by stabilizing endothelial cells, reducing transcytosis, limiting ICAM-1 and VCAM-1 expression, regulating tight junction integrity, and producing neurotrophic factors such as nerve growth factor (NGF), brain-derived neurotrophic factor (BDNF), and neurotrophin-3 (NT-3) [93,104]. During neuroinflammation, pericytes respond to TNF-α, IL-1β, IL-6, LPS, and saturated fatty acids [93,105,106,107,108] activating the NF-κB and MAPK pathways [93,107,108]. This activation induces production of IL-6, IL-8, CCL2, CCL5, CXCL10, and IL-1β—the latter through a non-canonical NLRP3-independent mechanism [106,107,109]. Pericyte activation also increases ICAM-1, VCAM-1, and MMP-9 expression through NF-κB and MAPK signaling, contributing to extracellular matrix degradation, BBB disruption [107,108,109,110], and enhanced leukocyte recruitment into the CNS, thereby perpetuating neuroinflammation.

Activation of these inflammatory pathways creates a highly oxidative microenvironment within the CNS [111]. As observed in adipose tissue, oxidative stress induces activation of NRF2, which is expressed in neurons, microglia, astrocytes, and BBB endothelial cells [112]. Beyond its antioxidant function, NRF2 suppresses glial activation and reduces the production of pro-inflammatory mediators [111,112,113,114]. Moreover, NRF2 contributes to the preservation of BBB integrity through antioxidant, anti-inflammatory, and tight junction–protective mechanisms [112]. Collectively, these processes promote persistent neuroinflammation associated with synaptic dysfunction, neuronal death, and cognitive impairment [57].

Under physiological conditions, synaptic pruning is an essential process for neural circuit remodeling [115]; however, excessive or dysregulated pruning has been associated with cognitive decline [115,116]. Persistent release of inflammatory cytokines, particularly TNF-α and IL-1β, also contributes to excitotoxicity by altering ion channel activity and synaptic receptor function [116]. IL-1β enhances N-methyl-D-aspartate (NMDA) receptor activity through Src kinase activation, increasing intracellular calcium influx and promoting excitotoxic neuronal injury [117]. Simultaneously, TNF-α increases α-amino-3-hydroxy-5-methyl-4-isoxazolepropionic acid (AMPA) receptor insertion while reducing γ-aminobutyric acid type A (GABA_A) receptor expression, thereby enhancing neuronal excitability and reducing inhibitory neurotransmission [118], ultimately promoting excitotoxicity [119]. Although physiological levels of TNF-α and IL-1β contribute to synaptic homeostasis and long-term potentiation (LTP), respectively, sustained activation of these cytokines during neuroinflammation results in cognitive dysfunction [116]. Elevated intracellular calcium further induces mitochondrial dysfunction and ROS generation, which in turn activates the NLRP3 inflammasome [120], amplifying inflammation and synaptic dysfunction [121]. Collectively, these structural and molecular alterations lead to dendritic spine loss, disruption of neuronal network architecture, and impairment of higher cognitive functions such as learning and memory [122]. Clinically, these mechanisms have been implicated in the development of neurodegenerative disorders, including Alzheimer’s and Parkinson’s diseases, as well as mood disorders such as depression and obesity-associated cognitive impairment [123].

## 5. Berberine in Obesity-Associated Neuroinflammation: Direct Evidence and Mechanistic Extrapolation Across Shared Inflammatory Pathways

Currently, few experimental studies have directly addressed the effect of berberine on the BBB in the context of obesity-induced neuroinflammation. For this reason, the available evidence has been organized according to its degree of relevance to this field. Direct studies are those that combine obesogenic conditions with measurements in the central nervous system; indirect metabolic studies are those derived from peripheral models; and indirect CNS studies are those derived from neurological disorders of non-obesogenic origin. In addition, molecular studies exploring mechanistic pathways such as TLR4/NF-κB/MAPK, ROS/NRF2, NLRP3/autophagy, or blood–brain barrier integrity were included. Although these studies are useful for supporting biological plausibility, they should not be considered equivalent to a demonstration of therapeutic efficacy in the specific model of obesity-associated neuroinflammation. This distinction is indicated for each study in Table 1.

BBR is considered a bioactive compound capable of modulating inflammatory and oxidative stress-related pathways in multiple tissues [17,19]. Compared to other natural compounds, BBR has shown favorable effects on obesity-associated metabolic parameters [134,135]. Furthermore, preclinical studies have reported the presence of BBR in brain tissue following systemic administration, supporting further investigation of its potential relevance to neurological and neurodegenerative disorders [19,136]. In rats, BBR was detected in brain tissue following an oral dose of 200 mg/kg, although its distribution was substantially greater in several peripheral organs [137]. In contrast, systemic exposure following oral administration in humans is very low. In a pharmacokinetic study involving 44 healthy individuals, a single oral dose of 1000 mg produced a median plasma Cmax of 0.434 ng/mL, with marked interindividual and sex-dependent variability [138]. CNS concentrations were not measured. Therefore, the evidence regarding the distribution of BBR to the brain is predominantly preclinical. The exposure gap between human pharmacokinetic data and several mechanistic experimental studies should also be considered when assessing translatability. The median plasma Cmax of 0.434 ng/mL observed after a 1000 mg oral dose in humans corresponds to approximately 1.3 nM BBR [138], whereas mechanistic studies summarized in Table 1 include in vitro concentrations of 10 μM in FFA-stressed Huh7 cells [8] and 1–10 μM in SH-SY5Y cells [139]. Thus, these nominal in vitro concentrations are several hundred to several thousand times higher than the reported median human peak plasma concentration. Although nominal cell-culture concentrations cannot be directly equated with in vivo plasma or CNS exposure because of differences in protein binding, intracellular accumulation, metabolites, and exposure duration, this substantial exposure gap limits direct translational inference. Future mechanistic studies should therefore incorporate exposure ranges that are better aligned with clinically achievable concentrations and, when possible, relate molecular effects to systemic and CNS pharmacokinetic measurements. Likewise, BBR exhibits poor aqueous solubility, limited intestinal absorption, presystemic degradation by the gut microbiota, extensive first-pass metabolism, and P-glycoprotein-mediated efflux. Collectively, these processes contribute to its low oral bioavailability. Consequently, several strategies have recently been developed to improve its pharmacokinetic profile [21,140,141] including polymeric nanoparticles, solid lipid nanoparticles, nanoemulsions, chitosan-based delivery systems [21], phospholipid complexes such as Phytosome^®^ [141], molecular hybrids with other bioactive compounds, and structural derivatives of BBR [140].

**Figure 3:**
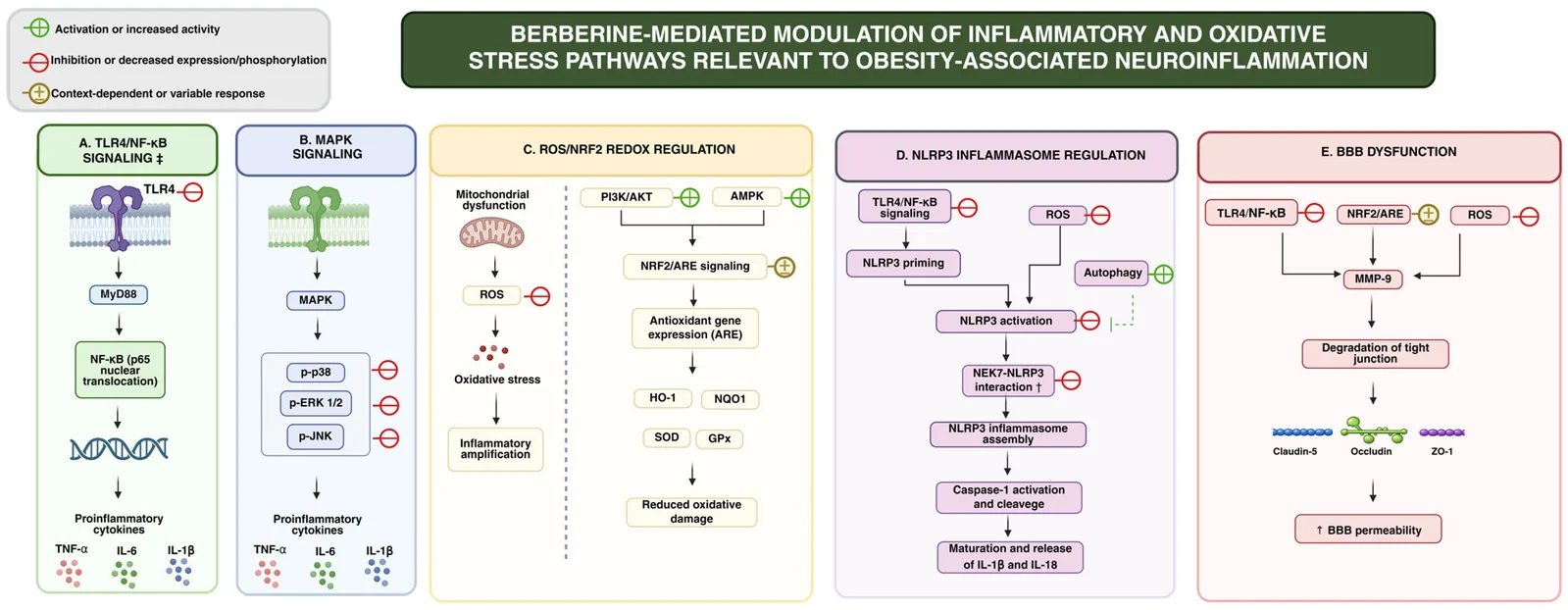
Berberine-mediated modulation of inflammatory and oxidative stress pathways relevant to obesity-associated neuroinflammation. (**A**) Berberine inhibits the TLR4/NF-κB signaling pathway by reducing TLR4 expression and interfering with intracellular signaling through disruption of the TLR4–MyD88 interaction. (**B**) Berberine suppresses MAPK signaling by reducing the phosphorylation of key MAPK family members, including p38 MAPK, ERK1/2, and JNK. (**C**) Berberine attenuates oxidative stress and modulates NRF2/ARE signaling in a context-dependent manner. Increased NRF2 expression, nuclear translocation, and/or transcriptional activity have been reported in several experimental models, whereas decreased NRF2 expression or activation has also been observed despite reduced ROS production. Therefore, NRF2 regulation should be interpreted as bidirectional and model-dependent rather than as a uniform activating effect of BBR. (**D**) Berberine inhibits NLRP3 inflammasome activation through multiple complementary mechanisms, including attenuation of ROS production, inhibition of TLR4/NF-κB signaling, promotion of autophagy, and disruption of NLRP3 inflammasome assembly by interfering with the NEK7–NLRP3 interaction. (**E**) Berberine modulates molecular pathways involved in MMP-9-mediated blood–brain barrier (BBB) disruption through inhibition of TLR4/NF-κB signaling, context-dependent modulation of NRF2/ARE signaling, and reduction in oxidative stress, thereby contributing to the preservation of BBB integrity. ‡ Inhibition of the TLR4–MyD88 interaction was demonstrated in experimental models using peritoneal macrophages. † Inhibition of the NEK7–NLRP3 interaction was demonstrated using THP-1 macrophages and mouse bone marrow-derived macrophages.

Despite these pharmacokinetic limitations, experimental evidence suggests that BBR can modulate multiple signaling pathways involved in obesity and neuroinflammation (Table 1; Figure 3) [17,19]. In this regard, studies conducted in several cell types have demonstrated that BBR can modulate TLR4 signaling through different mechanisms, either by reducing receptor expression [21,141,142,143,144,145,146,147] or by interfering with downstream intracellular signaling [148]. As mechanistic evidence in peripheral immune cells, co-immunoprecipitation assays in peritoneal macrophages demonstrated that BBR reduces the interaction between TLR4 and MyD88, leading to reduced NF-κB activation, decreased levels of pro-inflammatory cytokines, and reduced polarization toward the M1 phenotype, according to the classification used by the authors [148]. In contrast, as mechanistic support in the context of lipotoxicity, an experimental model of high-fat diet-induced liver disease showed that BBR reduced TLR4/MyD88/NF-κB expression, accompanied by decreased production of TNF-α, IL-6, and IL-1β [6]. Similarly, although in a different neuroinflammatory context, BBR was shown to reduce BBB permeability in a model of traumatic brain injury through inhibition of the TLR4/MyD88/NF-κB pathway, which promoted decreased MMP-9 and ICAM-1 expression. However, no significant restoration of tight junction proteins was observed, suggesting that the effect of BBR was predominantly anti-inflammatory rather than directly related to structural repair [124].

In addition, several studies in neuroinflammatory contexts have shown that BBR is associated with changes in the activation of proteins belonging to the MAPK family [10,125]. Of particular relevance to the axis analyzed in this review, in a model of obesogenic diet-induced metabolic alterations associated with hypothalamic inflammation, BBR decreased p38 MAPK and ERK1/2 phosphorylation in association with reduced NF-κB activation and changes in microglial polarization markers toward an anti-inflammatory profile [10]. In the same study, pharmacological inhibition of p38 MAPK with SB203580 reproduced several of the effects observed with BBR, including decreased IL-1β, IL-6, and TNF-α, increased Arg-1, and reduced NF-κB activation, providing mechanistic evidence for the involvement of p38 MAPK in these effects [10]. Consistently, although in a different neuroinflammatory context, BBR decreased the phosphorylation of p38 MAPK, ERK, and JNK in a model of LPS-induced neuroinflammation, in association with reduced TNF-α, IL-1β, and IL-6 levels and promotion of an anti-inflammatory phenotype [125]. Neuronal and synaptic preservation was also observed in this model, accompanied by improvements in neuronal plasticity and spatial memory [125]. Taken together, these findings support the involvement of MAPK signaling in the anti-inflammatory effects associated with BBR, particularly based on the evidence obtained through pharmacological inhibition of p38 MAPK [10]. However, they do not establish that MAPK modulation alone accounts for these effects or determine whether BBR acts directly on this pathway, given that concomitant changes in other inflammatory signaling cascades were also observed [10,125].

BBR has been reported to reduce oxidative stress in several experimental models, and NRF2/ARE signaling has been identified as a potential component of this response [139]. The PI3K/Akt pathway is among the proposed mechanisms underlying NRF2 regulation by BBR. However, the evidence supporting this relationship comes from neurological models unrelated to obesity. In a model of 6-OHDA-induced neurotoxicity, BBR treatment increased PI3K and Akt phosphorylation, accompanied by increased NRF2 expression and nuclear translocation and HO-1 expression following BBR treatment. Furthermore, pharmacological inhibition of PI3K/Akt with LY294002 attenuated these changes and the neuroprotective effects of BBR, supporting the involvement of this pathway in NRF2/HO-1 regulation within this experimental model [7]. Consistently, as indirect mechanistic evidence from a non-obesity neurological model, experimental studies in human dopaminergic cells exposed to oxidative stress showed that BBR activated PI3K/Akt and p38 signaling, promoting NRF2 translocation, increasing antioxidant response element (ARE)-dependent transcriptional activity, and upregulating heme oxygenase-1 (HO-1). These changes were associated with reduced oxidative stress, attenuation of neuronal apoptosis, and increased neuronal survival [126].

Another mechanism potentially involved in the regulation of NRF2 by BBR involves AMPK signaling. In a model of peripheral metabolic inflammation that included obese db/db mice, adipose tissue, and macrophages, BBR increased AMPK phosphorylation and decreased several pro-inflammatory responses. Importantly, pharmacological inhibition of AMPK with Compound C and the expression of a dominant-negative form of AMPK attenuated the effects of BBR, supporting the involvement of an AMPK-dependent anti-inflammatory mechanism in macrophages [149]. However, this study did not evaluate NRF2 or effects on the CNS and therefore constitutes indirect mechanistic evidence in the context of obesity-associated neuroinflammation. More directly related to the AMPK/NRF2 axis, although in a neurological context unrelated to obesity, Ma et al. used human SH-SY5Y neuronal cells with erastin-induced ferroptosis and a rat model of spinal cord injury. In both models, BBR treatment was associated with increased AMPK phosphorylation and increased NRF2 and HO-1 expression. Furthermore, pharmacological inhibition of AMPK with Compound C blocked the neuroprotective effects of BBR and attenuated the changes in NRF2 and HO-1, mechanistically supporting the involvement of AMPK in the BBR-mediated regulation of the NRF2/HO-1 axis within this experimental model [150].

However, indirect evidence from CNS models also suggests that NRF2-related signaling may contribute to the ability of BBR to protect the BBB. In a rat model of bilateral common carotid artery occlusion used to induce chronic cerebral hypoperfusion, BBR treatment activated the ERK/NRF2 pathway and reduced MMP-9 expression. These effects were associated with the protection of the BBB and hippocampal neurons, together with improved cognitive function [127].

Evidence from lipid accumulation models demonstrates the context-dependent nature of NRF2 regulation by BBR. In a murine model of high-fat diet-induced non-alcoholic fatty liver disease, BBR increased SOD activity and was accompanied by increased expression of NRF2, HO-1, and NQO1 [128]. In contrast, another study using in vivo and in vitro models of hepatic lipotoxicity reported reduced ROS production despite decreased NRF2 and HO-1 expression following BBR treatment [8]. The authors proposed that the reduction in NRF2/HO-1 reflected decreased compensatory antioxidant activation secondary to lower oxidative stress; however, this interpretation was not directly established, and alternative explanations related to the experimental model, basal redox status, treatment conditions, or the temporal dynamics of NRF2 signaling cannot be excluded.

Taken together, these findings indicate that the direction of change in NRF2 expression is not a consistent indicator of the antioxidant effect of BBR. Increased NRF2 expression or activation may be accompanied by improved redox homeostasis in some experimental settings, whereas oxidative stress may also be reduced without an increase in NRF2 expression [8,126,127,128].

Therefore, NRF2 expression alone should not be interpreted as a reliable indicator of antioxidant efficacy. Functional redox parameters, including ROS levels, the activity of antioxidant enzymes such as SOD and GPx, glutathione status, and oxidative damage, should be considered alongside NRF2 expression or transcriptional activity. In CNS- and BBB-related models, functional outcomes such as neuronal survival, MMP-9 activity, tight-junction integrity, and BBB permeability provide additional evidence of biological relevance [126,127].

Experimental evidence further suggests that BBR can suppress NLRP3 inflammasome activation [151,152]. This effect may occur through at least three mechanisms: restoration of autophagy, reduction in oxidative stress, and inhibition of inflammasome assembly [9,11,129,130,153]. Regarding autophagy, it is important to emphasize that increased AMPK phosphorylation alone should not be interpreted as evidence of an AMPK-dependent causal relationship. Studies in which this pathway was experimentally altered provide stronger mechanistic support. For example, pharmacological inhibition of AMPK, inhibition of autophagy, and Beclin-1 knockdown attenuated the effects of BBR on palmitate-induced NLRP3 activation, supporting the involvement of an AMPK-dependent autophagic mechanism in peripheral macrophages. Nevertheless, this evidence derives from peripheral metabolic inflammation and therefore remains indirect with respect to obesity-associated neuroinflammation [11]. As complementary mechanistic support, although not specific to BBR, AMPK-dependent regulation of autolysosome biogenesis in an experimental model of diabetic retinopathy [154]. Because this study evaluated arginyl–fructosyl–glucose rather than BBR, its findings support the biological plausibility of AMPK-dependent autolysosomal regulation but do not constitute evidence of a BBR-specific effect. As indirect CNS evidence from a model of neuroinflammation associated with Parkinson’s disease, BBR increased BECN1 expression, LC3-II levels, and autophagosome formation, while decreasing the expression of NLRP3, ASC, caspase-1, and IL-1β. Furthermore, the use of 3-MA to inhibit autophagy reduced the effects observed with BBR, suggesting that autophagy participates in the modulation of the NLRP3 inflammasome in this model [129]. Taken together, these findings support the restoration of autophagy as a potential mechanism through which BBR could modulate NLRP3-mediated inflammatory responses; however, the available evidence derives from peripheral models or neurological contexts unrelated to obesity.

BBR may also modulate NLRP3 activation by reducing oxidative stress [9,153]. As indirect evidence from a metabolic model, this mechanism was demonstrated in a model of lipotoxicity-induced steatohepatitis, in which BBR administration reduced ROS, thioredoxin-interacting protein (TXNIP), TNF-α, and NF-κB p65 phosphorylation, together with lower expression of NLRP3, caspase-1, and the cleaved *N*-terminal fragment of gasdermin D (GSDMD-N), a key executor of pyroptosis [9]. Importantly, BBR may not be limited to the indirect modulation of upstream signals but may also act directly on NLRP3 inflammasome assembly. As strong but indirect molecular evidence, a study using THP-1 cells, bone marrow-derived macrophages, and mouse models, BBR was shown to bind directly to NIMA-related kinase 7 (NEK7), thereby interfering with the NEK7–NLRP3 interaction, an essential step in inflammasome assembly. This molecular blockade reduced ASC oligomerization, caspase-1 activation, and IL-1β release independently of the NF-κB and TLR4 pathways [130].

Indirect evidence from a CNS model suggests that modulation of the ROS/NLRP3 axis may also contribute to the neuroprotective effects of BBR and preservation of BBB integrity. This was observed in a model of thioacetamide-induced hepatic encephalopathy, in which BBR reduced ROS, high-mobility group box 1 (HMGB1), NF-κB, and MAPK signaling, together with their associated cytokines. These effects were accompanied by preservation of the tight junction proteins ZO-1, occludin, and claudin-1, as well as reduced glial activation and decreased markers associated with NLRP3 inflammasome activation and pyroptosis [131].

Furthermore, indirect evidence from CNS models also links BBR treatment to the regulation of MMP-9 and the preservation of BBB integrity [132,133]. In an experimental model of intracerebral hemorrhage induced by autologous blood injection into the basal ganglia of C57BL/6 mice, BBR partially restored the expression of tight junction proteins, including ZO-1, occludin, and claudin-5. This effect was accompanied by reduced MMP-9 expression and decreased Evans blue extravasation. These findings were associated with lower levels of pro-inflammatory cytokines and activation of the AMPK/peroxisome proliferator-activated receptor gamma coactivator 1-alpha (PGC-1α) pathway [132]. Similarly, in a mouse model of autoimmune encephalitis, BBR reduced MMP-9 expression without significantly affecting MMP-2, suggesting that its effects may preferentially target MMP-9 [133].

However, although these preclinical models provide evidence of the potential effects of BBR on the CNS and BBB, their specific applicability to obesity-associated neuroinflammation remains limited. In contrast to the breadth of experimental evidence, the available studies in humans with obesity are more limited and have focused primarily on metabolic outcomes and peripheral inflammatory markers, without directly evaluating neuroinflammation. In adults with obesity, Bandala et al. (2024) [134] reported that BBR treatment was associated with changes in body composition, blood pressure, and peripheral adipokine expression. However, one of the study’s limitations is the lack of a direct relationship with neuroinflammation [134]. The available clinical evidence on BBR in obesity has focused mainly on anthropometric, metabolic, and peripheral inflammatory outcomes rather than direct measures of CNS inflammation [16,134].

This distinction is important because peripheral inflammatory biomarkers do not necessarily reflect inflammatory activity within the CNS. A systematic review and meta-analysis of paired blood and cerebrospinal fluid samples showed only a low overall correlation between peripheral and central inflammatory markers (pooled r = 0.21), suggesting that circulating inflammatory biomarkers are unreliable indicators of the CNS neuroinflammatory profile [155]. Therefore, current human evidence supports the metabolic and peripheral anti-inflammatory effects of BBR but does not establish a direct therapeutic effect on obesity-associated neuroinflammation. Consequently, peripheral inflammatory biomarkers should be extrapolated to CNS inflammation with caution.

The overall interpretation of these findings should also consider the methodological heterogeneity among studies, particularly differences in the species or cell types used, BBR doses and concentrations, routes of administration, treatment duration, and outcomes evaluated. These differences hinder the direct comparison of results and may contribute to variability in the magnitude or direction of the observed effects. Therefore, the convergence of findings regarding specific molecular pathways should be interpreted in light of the characteristics and limitations of each experimental model.

In conclusion, the accumulated evidence suggests that BBR may act as a modulator of both obesity-induced chronic low-grade systemic inflammation and neuroinflammation [17,19]. Experimental studies indicate that these effects include reduced glial activation, preservation of BBB integrity, attenuation of neuronal damage, and improvement in cognitive function. Nevertheless, these benefits may arise indirectly from modulation of the neuroinflammatory and oxidative microenvironment rather than from direct neuroprotective action at the neuronal level [124].

## 6. Conclusions and Future Perspectives

Taken together, the available evidence provides mechanistically based support suggesting that BBR may modulate relevant pathways involved in obesity-associated neuroinflammation, including the TLR4/NF-κB/MAPK, ROS/NRF2, and NLRP3/autophagy axes. However, direct evidence from models that simultaneously integrate obesogenic conditions and CNS neuroinflammatory alterations remains limited; therefore, the reported effects should be interpreted as evidence of biological potential rather than established therapeutic efficacy. Considering the growing impact of neuroinflammation as a consequence of obesity and other associated comorbidities, identifying feasible therapeutic alternatives is a priority. In this regard, future research should prioritize pharmacokinetic and pharmacodynamic studies in humans designed to determine whether clinically relevant oral doses of BBR produce measurable exposure in the CNS and interact with molecular targets, including paired plasma and CSF measurements or other validated approaches to assess CNS exposure when feasible. Controlled clinical trials in obese populations should incorporate CNS-relevant neuroinflammatory endpoints related to the BBB and cognitive endpoints, rather than relying exclusively on metabolic or peripheral inflammatory biomarkers. Concurrently, diet-induced obesity models should directly compare native BBR with enhanced-bioavailability formulations, including nanoformulations, using direct comparative designs that assess systemic exposure, brain distribution, activation of signaling pathways, blood–brain barrier integrity, and cognitive outcomes. Such studies would help determine whether the mechanistic effects observed at relatively high preclinical exposure levels can be replicated under clinically relevant exposure conditions.

## Figures and Tables

**Table 1:** Preclinical evidence of the effects of BBR in models of neuroinflammation and obesity.

Reference	Experimental Model and BBR Exposure	Main Mechanistic and Functional Findings	Relevance to Obesity-Associated Neuroinflammation and Main Limitation
Wang et al., 2020 [6]	HFD-induced NAFLD in rats; 200 mg/kg/day, oral, 12 weeks.	TLR4/MyD88/NF-κB: lower TLR4, MyD88 and NF-κB expression/nuclear translocation; lower TNF-α, IL-6 and IL-1β; improved hepatic inflammation.	Indirect—metabolic. Peripheral liver model with no CNS outcomes; pathway changes support association but do not establish TLR4 dependence.
Chen et al., 2014 [124]	Controlled cortical impact TBI in mice and complementary glial cultures; 10 mg/kg i.p., 10 min after injury; delayed 3 h treatment also evaluated.	TLR4/MyD88/NF-κB–BBB: lower glial activation, inflammatory mediators, MMP-9, ICAM-1, edema and BBB permeability; claudin-5 and ZO-1 were not restored.	Indirect—CNS. Acute traumatic injury, parenteral exposure and pre-existing BBB disruption; etiologically unrelated to obesity.
Feng et al., 2025 [10]	HFHFD-fed mice; complementary BV2 microglia and neuronal co-culture experiments; oral BBR for 4 weeks at 200 mg/kg/day (high-dose group) and 100 mg/kg/day (low-dose group).	MAPK/NF-κB: lower IKKβ/NF-κB, p38 and ERK1/2 phosphorylation and inflammatory signaling. p38 inhibition provided additional evidence for MAPK involvement.	Direct. Strongest alignment with obesity-associated hypothalamic neuroinflammation. Preclinical; original phenotyping relies partly on the M1/M2 framework.
Jiang et al., 2026 [125]	Male C57BL/6J mice with intrahippocampal LPS; 50 or 100 mg/kg/day by gavage for 21 days; complementary BV2/neuronal experiments.	MAPK: lower JNK, ERK1/2 and p38 phosphorylation, Iba1/iNOS-associated inflammatory features and cytokines; increase Arg1-associated features; preserved synaptic proteins, LTP and spatial memory.	Indirect—CNS. Direct neuroinflammation evidence but induced by LPS, not obesity. MAPK inhibition is demonstrated, but upstream causality remains unresolved.
Sun et al., 2017 [8]	HFD C57BL/6 mice (300 mg/kg/day orally, 8 weeks), HFD SD rats (200 mg/kg/day by gavage, 16 weeks) and FFA-stressed Huh7 cells (10 μM; 24 h).	ROS/NRF2: lower cytoplasmic and mitochondrial ROS while reducing compensatory NRF2 nuclear translocation and HO-1 activation under lipid stress.	Indirect—metabolic. Hepatic models; no CNS/BBB outcomes. Important evidence that NRF2 directionality is context dependent.
Bae et al., 2013 [126]	Human SH-SY5Y dopaminergic cells exposed to 6-OHDA; BBR 1–10 μM, with 12 h pretreatment.	PI3K/Akt–p38–NRF2/HO-1: increase NRF2 nuclear translocation, ARE activity and HO-1; lower ROS and apoptosis. siNRF2 and kinase inhibitors supported pathway involvement.	Indirect—CNS; strong mechanistic support. Neurotoxin model in an immortalized cell line; no obesity component.
Wang et al., 2022 [127]	Rat bilateral carotid artery occlusion/2VO model of chronic cerebral hypoperfusion; 90 mg/kg p.o. for 42 days.	ERK/NRF2–BBB: increase ERK/NRF2 signaling; lower VEGF-A and MMP-9; preserved BBB and hippocampal neurons and improved cognition.	Indirect—CNS. Cerebral hypoperfusion rather than obesity; pathway activation is associated with protection but necessity was not demonstrated.
Deng et al., 2019 [128]	HFD-induced NAFLD in rats; 100 mg/kg/day by gastrogavage for 8 weeks.	NRF2/ARE: increase SOD and GSH, lower MDA and increase NRF2/ARE-related antioxidant expression; improved hepatic steatosis.	Indirect—metabolic. Liver model without CNS endpoints; NRF2 dependence was not experimentally tested.
Mai et al., 2020 [9]	MCD diet-induced NASH in mice and complementary AML12 hepatocytes; 100 mg/kg/day by gavage during the final 2 weeks of the 5-week model.	ROS/TXNIP/NLRP3: lower ROS, TXNIP, NF-κB, NLRP3, caspase-1 and GSDMD-mediated pyroptosis.	Indirect—metabolic. Hepatic model; the MCD model does not reproduce obesity and insulin resistance adequately; no CNS endpoints.
Zhou et al., 2017 [11]	HFD-fed C57BL/6 mice; 75 or 150 mg/kg by gavage during the final 3 weeks of a 14-week HFD; BMDMs and THP-1 macrophages in vitro.	AMPK–autophagy–NLRP3: lower NLRP3/caspase-1/IL-1β and mitochondrial ROS; increase autophagy. Beclin-1 knockdown, 3-MA and AMPK inhibition attenuated BBR effects.	Indirect—metabolic; strong mechanistic support. Demonstrates pathway dependence, but in peripheral macrophages rather than CNS cells.
Huang et al., 2021 [129]	MPTP-induced Parkinsonian mice and MPP+-stimulated BV2 cells; 50 mg/kg intragastrically once daily for 21 days in vivo.	Autophagy–NLRP3: increase autophagic activity and lower NLRP3, PYCARD, cleaved caspase-1 and IL-1β. Autophagy inhibition with 3-MA attenuated BBR effects.	Indirect—CNS; mechanistic support. Neurotoxin/PD model rather than obesity-associated neuroinflammation.
Zeng et al., 2021 [130]	THP-1 macrophages, mouse BMDMs and inflammatory mouse models; BBR evaluated across micromolar exposures; direct NEK7 target engagement, KD = 15.6 µM and IC50 = 4.2 µM.	NEK7–NLRP3: activity-based protein profiling demonstrated direct BBR binding to NEK7 at R121, disrupting NEK7–NLRP3 interaction and reducing inflammasome assembly/IL-1β independently of TLR4/NF-κB.	Indirect—molecular; very strong mechanistic evidence. Direct target-level causality, but not demonstrated specifically in obesity-associated CNS inflammation.
Ali and Datusalia, 2025 [131]	TAA-induced acute hepatic encephalopathy in rats; BBR 10, 30, or 100 mg/kg (*n* = 10/group); lactulose 8 mL/kg as standard-drug control.	ROS/NLRP3–BBB: lower ROS/HMGB1/NF-κB/NLRP3 signaling, glial activation and pyroptotic markers; preservation of tight-junction proteins and BBB integrity.	Indirect—CNS. Hepatic encephalopathy/hyperammonemia rather than obesity.
Wu et al., 2022 [132]	Autologous-blood intracerebral hemorrhage in mice; 50, 100 or 200 mg/kg by oral gavage at 1, 12, 24, 48 and 72 h after ICH.	AMPK/PGC-1α–BBB: lower inflammatory cytokines, MMP-9, edema and BBB leakage; restored ZO-1, occludin and claudin-5; increase AMPK/PGC-1α signaling.	Indirect—CNS. Acute hemorrhagic injury and relatively high experimental exposures; AMPK dependence was not established.
Ma et al., 2010 [133]	MOG-induced EAE in female C57BL/6 mice; 30 mg/kg p.o. daily from disease onset until day 35 post-immunization.	BBB/MMP-9: lower BBB permeability, inflammatory infiltration and MMP-9 expression/activity; MMP-2 was not significantly modified.	Indirect—CNS. Autoimmune demyelinating model, not metabolic or obesity-associated neuroinflammation.
Bandala et al., 2024 [123]	50 adults with obesity; BBR arm *n* = 25; 500 mg orally three times daily before meals for 3 months.	lower body weight and selected adiposity/BP measures; lower peripheral visfatin and apelin expression and increase omentin expression.	Human—indirect. Provides obesity-specific clinical evidence, but no CNS, BBB, neuroimaging or CNS-specific inflammatory endpoints; small active-comparator study without placebo.

## Data Availability

No new data were created or analyzed in this study. Data sharing is not applicable to this article.

## Abbreviations

AKT	Protein Kinase B
ALT	Alanine Aminotransferase
AMPK	AMP-Activated Protein Kinase
ARE	Antioxidant Response Element
ASC	Apoptosis-Associated Speck-Like Protein Containing a CARD
AST	Aspartate Aminotransferase
BBB	Blood–Brain Barrier
BBR	Berberine
BDNF	Brain-Derived Neurotrophic Factor
CCL2	C-C motif chemokine ligand 2
CCL5	C-C motif chemokine ligand 5
CT	Total Cholesterol
CXCL10	C-X-C Motif Chemokine Ligand 10
DAMPS	Damage-Associated Molecular Patterns
ERK1/2	Extracellular Signal-Regulated Kinases 1 and 2
FFA	Free Fatty Acids
GPX4	Glutathione Peroxidase 4
GSH	Reduced Glutathione
GSDMD	Gasdermin D
HFD	High-Fat Diet
HFHFD	High-Fat High-Fructose Diet
HIF-1α	Hypoxia-Inducible Factor 1 Alpha
HMGB1	High-Mobility Group Box 1
HO-1	Heme Oxygenase-1
ICAM-1	Intercellular Adhesion Molecule 1
IKKβ	IκB Kinase Beta
IL-1β	Interleukin-1 Beta
IL-1R	Interleukin-1 Receptor
IL-6	Interleukin-6
IL-6R	Interleukin-6 Receptor
IL-18	Interleukin-18
iNOS	Inducible Nitric Oxide Synthase
JNK	*c*-Jun *N*-terminal Kinase
KEAP1	Kelch-Like ECH-Associated Protein 1
LC3-II	Microtubule-Associated Protein 1 Light Chain 3-II
LDL-C	Low-Density Lipoprotein Cholesterol
LPS	Lipopolysaccharide
LTP	Long-Term Potentiation
MAPK	Mitogen-Activated Protein Kinase
MCP-1	Monocyte Chemoattractant Protein-1
MDA	Malondialdehyde
MMP-2	Matrix Metalloproteinase-2
MMP-9	Matrix Metalloproteinase-9
MyD88	Myeloid Differentiation Primary Response 88
NAFLD	Non-Alcoholic Fatty Liver Disease
NASH	Non-Alcoholic Steatohepatitis
NEK7	NIMA-Related Kinase 7
NF-κB	Nuclear Factor Kappa B
NGF	Nerve Growth Factor
NLRP3	NOD-, LRR- and Pyrin Domain-Containing Protein 3
NMDA	*N*-Methyl-D-Aspartate Receptor
NQO1	NAD(P)H Quinone Oxidoreductase 1
NRF2	Nuclear Factor Erythroid 2-Related Factor 2
NT-3	Neurotrophin-3
PI3K	Phosphoinositide 3-Kinase
ROS	Reactive Oxygen Species
SHH	Sonic Hedgehog
SOD	Superoxide Dismutase
STAT	Signal Transducer and Activator of Transcription
STZ	Streptozotocin
TGF-β	Transforming Growth Factor Beta
TLR2	Toll-Like Receptor 2
TLR4	Toll-Like Receptor 4
TNF-α	Tumor Necrosis Factor Alpha
TNFR	Tumor Necrosis Factor Receptor
TRIF	TIR-domain-containing adapter-inducing interferon-β.
TXNIP	Thioredoxin-Interacting Protein
VAT	Visceral Adipose Tissue
VCAM-1	Vascular Cell Adhesion Molecule 1
ZO-1	Zonula Occludens-1
ZO-2	Zonula Occludens-2
ZO-3	Zonula Occludens-3
